# Identification of Metabolic Signature Associated with Idiopathic Inflammatory Myopathy Reveals Polyamine Pathway Alteration in Muscle Tissue

**DOI:** 10.3390/metabo12101004

**Published:** 2022-10-21

**Authors:** Jihyun Kang, Jeong Yeon Kim, Youjin Jung, Seon Uk Kim, Eun Young Lee, Joo-Youn Cho

**Affiliations:** 1 Department of Clinical Pharmacology and Therapeutics, Seoul National University College of Medicine and Hospital, Seoul 03080, Korea; 2 Department of Biomedical Sciences, Seoul National University College of Medicine, Seoul 03080, Korea; 3 Division of Cellular Genomics, GENOME INSIGHT Technologies, Seoul 06735, Korea; 4 Division of Rheumatology, Department of Internal Medicine, Seoul National University College of Medicine, Seoul 03080, Korea; 5 Division of Rheumatology, Department of Internal Medicine, Seoul Metropolitan Seoul Medical Center, Seoul 02053, Korea

**Keywords:** idiopathic inflammatory myopathy, biomarker, polyamine pathway

## Abstract

Idiopathic inflammatory myopathy (IIM) is hard to diagnose without a muscle biopsy. We aimed to identify a metabolite panel for IIM detection by metabolomics approach in serum samples and to explore the metabolomic signature in tissue samples from a mouse model. We obtained serum samples from IIM patients, ankylosing spondylitis (AS) patients, healthy volunteers and muscle tissue samples from IIM murine model. All samples were subjected to a targeted metabolomic approach with various statistical analyses on serum and tissue samples to identify metabolic alterations. Three machine learning methods, such as logistic regression (LR), support vector machine (SVM), and random forest (RF), were applied to build prediction models. A set of 7 predictive metabolites was calculated using backward stepwise selection, and the model was evaluated within 5-fold cross-validation by using three machine algorithms. The model produced an area under the receiver operating characteristic curve values of 0.955 (LR), 0.908 (RF) and 0.918 (SVM). A total of 68 metabolites were significantly changed in mouse tissue. Notably, the most influential pathways contributing to the inflammation of muscle were the polyamine pathway and the beta-alanine pathway. Our metabolomic approach offers the potential biomarkers of IIM and reveals pathologically relevant metabolic pathways that are associated with IIM.

## 1. Introduction

Idiopathic inflammatory myopathy (IIM) is a rare inflammatory disease characterized by immune-mediated myositis that leads to progressive proximal muscle weakness and is accompanied by various extramuscular manifestations, including skin rash, arthritis, and interstitial lung disease [1]. The incidence and prevalence of IIM remain unclear, but a systemic review reported the incidence as ranged from 1.16 to 19/million/year and its prevalence ranged from 2.4 to 33.8 per 100,000 inhabitants [2]. Moreover, the incidence of IIM is gradually increasing, which may reflect the advances in diagnostic technologies.

In 1975, Bohan and Peter suggested the diagnostic criteria of IIM, especially polymyositis (PM) and dermatomyositis (DM), which is predominantly used despite several limitations [3,4]. The recent discovery of myositis-specific auto-antibodies (MSAs) and myositis-associated auto-antibodies (MAAs), which are expressed in up to 60% of IIM patients, have majorly advanced the diagnosis of IIM [5]. However, the disadvantages of this technique include its lack of wide availability and the fact that approximately 40% of IIM patients are antibody-negative [6]. Therefore, a muscle biopsy may be necessary if the diagnosis cannot be confirmed on the basis of clinical and laboratory findings. However, histopathologic findings are often indefinite for the diagnosis because of the patchy distribution of inflammatory foci in muscle tissue or due to the use of medication prior to the pathologic diagnosis.

C protein-induced myositis (CIM) is a murine model of polymyositis (PM) that is induced by a single immunization with recombinant skeletal muscle C protein fragments in a C57BL/6 mouse. Infiltration of CD8+ T cells is the primary mechanism of muscle injury, and increased levels of interleukin (IL)-1, IL-6, and tumor necrosis factor (TNF)-α are crucial indications for the development of CIM. Consequently, the CIM model is used to study the pathology of PM for its comparable disease mechanism [7,8,9].

Previous studies reported genetic and environmental risk factors associated with IIM, including muscle weakness and damage to immune and non-immune mechanisms [10]. The metabolome, a set of metabolites, can be applied to discover biomarkers and describe interactions among genotype, diet, and environmental factors to elucidate the molecular mechanisms in IIM [11]. Furthermore, tissue metabolomics allows informing the localized effects of environmental factors and complex interactions that occur at the direct site of pathogenesis [12]. Metabolic alterations have been reported in IIM patients, describing metabolic dysregulation in serum and muscle tissues [13]. Key metabolic pathways, including anaerobic metabolism, oxidative defect, and muscle catabolism, were evaluated through metabolomics [13]. However, none of the previous studies remained serum biomarkers using metabolomics to classify IIM or aid in diagnosis. This study was conducted with the aim of identifying the metabolite panel as a potential biomarker for differentiating IIM from controls or disease controls and exploring the metabolic signature of IIM pathology by using a mouse model.

## 2. Materials and Methods

### 2.1. Study Participants

Patients were recruited from Seoul National University Hospital from March 2010 to February 2020. Patients with IIM who were 19 years or older and diagnosed according to probable or definite PM or DM based on the Bohan and Peter criteria were eligible for inclusion [3,4]. Patients with other rheumatic diseases were excluded. The reference group was composed of patients with ankylosing spondylitis (AS), and the healthy control group included participants without any immune-mediated disease. AS is a chronic inflammatory autoimmune disease that mainly affects spine joints, causing severe, chronic pain, and IIM is a chronic inflammatory disease of unknown etiology that may affect the skin, muscles, and lungs. These two autoimmune diseases have different etiopathogeneses as well as clinical and genetic characteristics. In this study, we compared and analyzed the serum of AS patients as a disease control. The diagnosis of AS was based on the 1984 Modified New York Criteria for AS.

We obtained serum samples of the participants once at the enrollment date. Information on demographics, including age, sex, and body mass index (BMI, kg/m^2^), was collected from all participants. In addition, the following information was collected from IIM patients: clinical manifestations at IIM onset, including proximal muscle weakness, skin rash, dyspnea, dysphagia, Raynaud’s phenomenon, and arthralgia; previous treatment history of glucocorticoids, intravenous immunoglobulin, or immunosuppressive agent use; laboratory results including white blood cell count, creatinine kinase, aldolase, myoglobin, lactate dehydrogenase, erythrocyte sedimentation rate, and C-reactive protein at the sampling date and positivity of antinuclear antibodies and anti-Jo-1 (anti-histidyl-transfer RNA synthetase antibody). The results of the diagnostic tests for myositis, including electromyography, muscle biopsy, chest computed tomography, and pulmonary function test, were also retrieved.

Written informed consent was obtained from all participants before their enrollment in the trial. This study was approved by the institutional review board of the Seoul National University Hospital (study identification number: g1103-151-357 and 1902-120-1013) and was conducted in accordance with the Declaration of Helsinki.

### 2.2. Induction of C Protein-Induced Myositis

C57BL/6 mice were purchased from OrientBio (Sungnam, Korea). Female mice (age 8 to 10 weeks) were immunized intradermally with 200 μg C-protein fragments emulsified in complete Freund’s adjuvant containing 100 μg heat-killed *Mycobacterium butyricum* (Difco, Franklin Lakes, NJ, USA) [7]. The immunogens were injected at multiple sites over the back and into the foot pads, and 250 ng pertussis toxin (Sigma-Aldrich, St. Louis, MO, USA) diluted with 0.03% Triton X was injected intraperitoneally. Mice were sacrificed on day 14 after immunization, and the sera and proximal muscles (hamstring and quadriceps) of both hind legs were harvested.

### 2.3. Histological Analysis

Hematoxylin and eosin-stained 10-μm sections of the proximal muscles were examined histologically for the presence of mononuclear cell infiltration and necrosis of muscle fibers. The histologic severity of inflammation in each muscle block was graded as Grade 1,  involvement of a single muscle fiber; Grade 2,  a lesion involving 2–5 muscle fibers; Grade 3,  a lesion involving 6–15 muscle fibers; Grade 4, a lesion involving 16–30 muscle fibers; Grade 5,  a lesion involving 31–100 muscle fibers; and Grade 6, a lesion involving >100 muscle fibers. When multiple lesions with the same grade were found in a single muscle section, an additional 0.5 points was added to the grade. The histologic grading was based on a method that was modified from that of Sugihara et al. [7]. All experiments were done under specific pathogen-free conditions. The animal experiment was approved by the Institutional Animal Care and Use Committee of Seoul National University Hospital [IACUC No. 15-0058-C1A1].

### 2.4. Mass Spectrometry-Based Targeted Metabolomics Assay

Metabolomics analysis of human and mouse samples was performed using the Biocrates AbsoluteIDQ p180 platform (Supplementary Figure S1). This high-throughput metabolome platform combines a flow-injection analysis and liquid chromatography method that enables the quantification of amino acids, acylcarnitines, sphingomyelins (SMs), lysophosphatidylcholines (lysoPCs), phosphatidylcholines (PCs), hexoses, and biogenic amines. These assays were analyzed using API 4000 QTRAP (AB Sciex, Framingham, MA, USA) equipped with an Agilent 1200 series high-performance liquid chromatography (HPLC) system (Agilent Technologies, Santa Clara, CA, USA) and an AB SCIEX 5500 QTrap mass spectrometer (AB Sciex, USA) equipped with a Waters ACQUITY ultra-performance liquid chromatography (UPLC) I-Class (Waters, Milford, MA, USA) with electrospray ionization. The concentration of each metabolite was measured in ng/mL. The calibration standards, internal standards, quality controls, and 10 µL of human and mouse samples were applied onto the 96-well extraction plate and dried under nitrogen gas. After derivatization, all metabolites were extracted for mass spectrometry analysis.

### 2.5. Data Processing and Normalization

Two batches, including all human and mouse samples, were normalized by MetIDQ software with standardized quality control to adjust the batch effect. After normalization, data were analyzed with MetaboAnalyst 5.0. A missing value refers to an observed value that is below the limit of detection or the absence of a metabolite. Metabolites with 50% missing values were excluded, and the remaining missing values were imputed with 1/5 of the minimum positive value of each variable.

### 2.6. Statistical Analysis

Multivariate, univariate, cluster and enrichment pathway analyses were performed in MetaboAnalyst 5.0. Significant metabolites were selected with a false discovery rate (FDR) adjusted *p*-value less than 0.05 by using the Kruskal-Wallis one-way analysis of variance (ANOVA) followed by Dunn’s post hoc test for human serum samples and by using the non-parametric t-test for mouse samples. Model and machine learning analyses were performed using R software (version 4.2.0). The number of significant metabolites was reduced by backward stepwise selection. A predictive model was established using logistic regression (LR), random forest (RF), and a support vector machine (SVM). The receiver operating characteristic (ROC) curve was used for the evaluation of model performance. Additionally, network analysis was processed with an FDR-adjusted *p*-value, fold change and fold change direction by using MetaMapp and was visualized using Cytoscape.

### 2.7. Protein Extraction and Western Blot Analysis

Skeletal muscles were extracted using a lysis buffer containing 20 mM Hepes-NaOH (pH 7.0), 0.15 M NaCl, 10% glycerol, 1% Nonidet P-40, 1 mM EDTA, 1 mM EGTA, 10 mM β-phosphoglycerate, 1 mM sodium vanadate, 5 mM NaF, 1 mM trichostatin A, and 20 mM nicotinamide, along with a mixture of protease inhibitor and phosphatase inhibitor (Sigma Aldrich, St. Louis, MO, USA). The lysates were centrifuged to remove debris, and the supernatants were used for immunoblot analysis. The final protein concentrations were determined using the Bradford protein assay (Bio-Rad, Hercules, CA, USA). The protein extracts were subjected to SDS-PAGE gel (8–15%), and NuPAGE Novex 4–12% Bis-Tris gel electrophoresis (Invitrogen) and electrophoresis were carried out using sodium dodecyl sulfate-10% polyacrylamide gels electrophoresis (SDS-PAGE). The separated proteins were transferred to a polyvinylidene difluoride membrane. The membrane was incubated overnight at 4 °C with primary antibodies (ODC-1 (Abcam), SMOX (Abcam), tubulin (cell signaling)) and then for 1 h at room temperature with horseradish peroxidase-conjugated secondary antibodies and enhanced chemiluminescence reagents (ELPIS Biotech, Daejeon, Korea). Films were scanned, and optical densities were quantified using ImageJ software.

### 2.8. ELISA

Serum cytokines were determined by using IL-6, IL-1β, and TNF-α, ELISA kits (R&D Systems, Minneapolis, MN, USA).

## 3. Results

### 3.1. Clinical Characteristics of the Participants

Between March 2010 and August 2019, 50 patients with IIM, 30 patients with AS, and 10 healthy controls were enrolled in the study. Baseline characteristics of the study population are shown in Table 1, and there was no statistically significant intergroup difference in age, sex, and BMI. IIM was diagnosed at a mean age of 48.4 years, and the mean disease duration was 2.24 years. Thirty-four patients (68%) presented with proximal muscle weakness, 30 (60%) had skin manifestations such as Gottron’s sign, heliotrope rash, or V-neck sign, and 27 (55.1%) were diagnosed with interstitial lung disease at an initial presentation. Anti-Jo-1 antibody was identified in six patients. Forty-one patients (82%) had a previous history of glucocorticoid therapy before the enrollment date. The mean values were stated as CK of 532 U/L, LDH of 329 IU/L, and CRP of 1.44 mg/dL at the sample collection date.

### 3.2. Metabolic Profiling of Healthy Control, AS and IIM Patients

**Figure 1:**
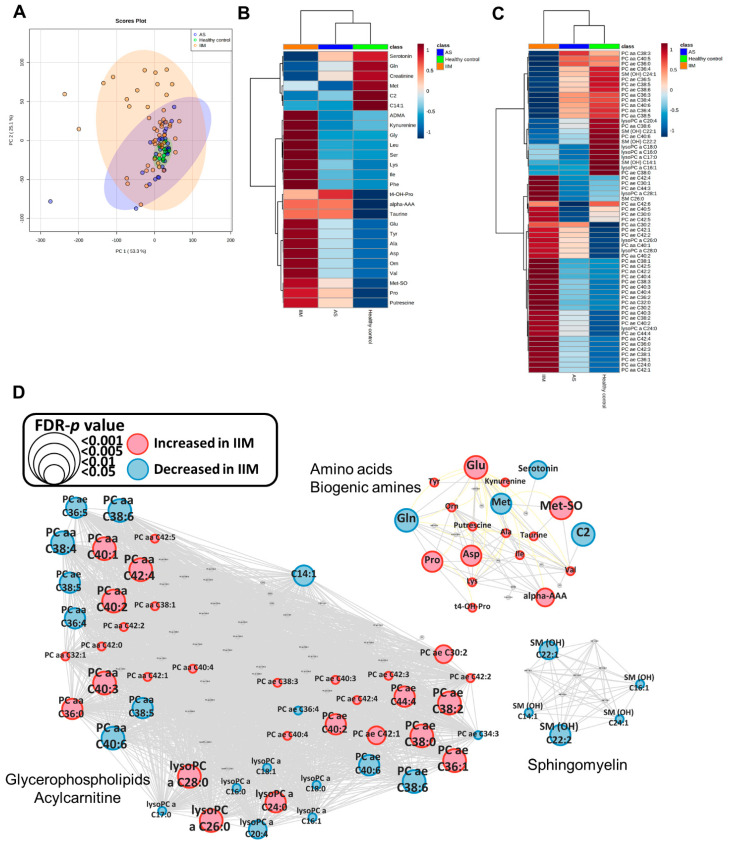
Metabolomic analysis of IIM patients, AS patients, and healthy controls. (**A**) PCA plot of AS patients, IIM patients, and healthy controls. (**B**) Heatmap of significant metabolites with group average in IIM, including amino acid, biogenic amine and acylcarnitine class. (**C**) Heatmap of significant metabolites with group average in IIM, including SMs, lysoPCs, PC, and hexoses. For the full name of metabolites, please see Supplementary Table S2. (**D**) MetaMapp metabolomic networks of human serum alterations in IIM patients compared to healthy controls. Border color and node color: red indicates up-regulation in IIM, and blue indicates down-regulation in IIM. Node size indicates the FDR-adjusted *p*-value.

We first sought to explore the metabolic signature of IIM in serum samples of IIM patients, AS patients and healthy controls. After missing value imputation, a total of 148 metabolites remained for further analysis in human serum. Principal component analysis (PCA) in human serum samples showed differences between the IIM and other groups (Figure 1A), whereas the AS and healthy control groups showed no intergroup difference. In total, 88 significant metabolites were identified using ANOVA with an FDR-adjusted *p*-value less than 0.05 for IIM, AS, and healthy control groups (Supplementary Table S1). The clustered heatmap using Euclidean distance and ward method shows different clusters with metabolite similarity (Supplementary Figure S2). The heatmap with group averages clearly showed differences between the IIM, AS, and healthy control groups (Figure 1B,C). In particular, the metabolic signature between healthy control and IIM was distinctively different. L-Acetylcarnitine, cis-5-tetradecenoylcarnitine, serotonin, glycine, creatinine and methionine were down-regulated, whereas all other significant metabolites were upregulated in IIM compared to healthy control (Figure 1B). To visualize the metabolomic data globally, mapping of biochemical pathways and chemical similarity for significant metabolites in IIM compared to healthy controls was conducted (Figure 1D). All significant metabolites were mapped as three main clusters: amino acids and biogenic amine, sphingomyelin, glycerophospholipids, and acylcarnitine. Clearly, all significantly changed sphingomyelin were down-regulated in IIM compared to the healthy controls. Besides sphingomyelin, numerous metabolites in other clusters were altered in the serum of IIM compared to healthy controls. The results support that inflammation in muscle impacts on systemic serum metabolite profile.

### 3.3. Predictive Biomarker and Machine Learning Algorithm Optimization for Distinguishing IIM

**Figure 2:**
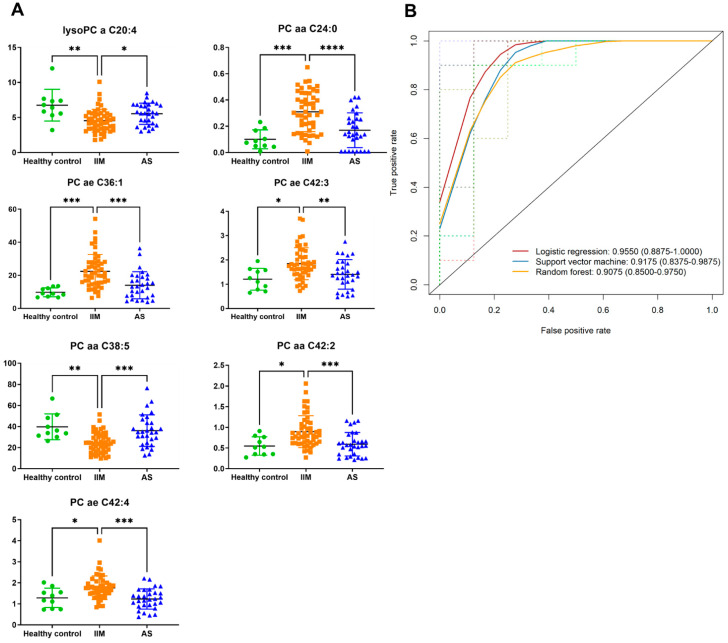
Serum metabolites as biomarkers for IIM. (**A**) The individual plot of IIM-specific metabolites was selected by the backward stepwise method for constructing a prediction model. * *p* < 0.05; ** *p* < 0.005; *** *p* < 0.0005, and **** *p* < 0.0001. (**B**) ROC curves for seven sets of metabolites with five-fold cross-validation. AUC values were calculated for each machine learning method; red indicates logistic regression, blue indicates support vector machine, and yellow indicates random forest.

Following the ANOVA test, multiple comparisons were performed to identify 37 IIM-specific metabolites (Supplementary Table S3). Among these IIM-specific metabolites, a combination of seven metabolites was calculated to distinguish IIM from AS and healthy controls using backward stepwise selection (Figure 2A). LR, RF, and SVM algorithms were used to establish a prediction model of IIM. An ROC analysis with LR, RF, and SVM was performed to assess model performance and showed an area under the ROC curve (AUC) values of 0.966 (95%CI: 0.928–1.000), 1 (1.000–1.000) and 0.957 (0.910–1.000), respectively (Supplementary Figure S3). The evaluation results of the prediction model using a five-fold cross-validation method are shown in Figure 2B. The AUC values were 0.955 (LR), 0.908 (RF) and 0.918 (SVM). By using the prediction model as well as 5-fold cross-validation, seven metabolite panels were identified as the powerful contributor to help distinguish IIM from other groups.

### 3.4. Metabolic Profiling in the C-Protein-Induced Myositis Mouse Model

**Figure 3:**
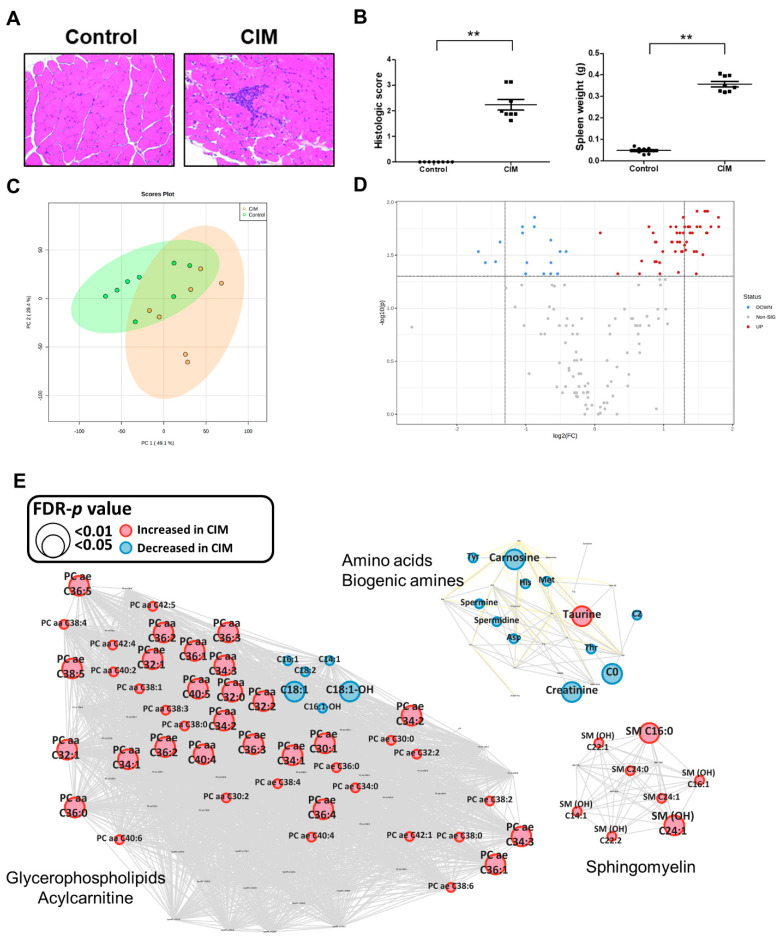
Effect of myositis on skeletal muscle tissue in the mouse model. (**A**) histology of control and CIM. (**B**) The histologic score of control and CIM. (** *p* < 0.0001) (**C**) PCA plot of control and CIM skeletal muscle tissue. (**D**) Volcano plot from *t*-test results of control and CIM. Red dots are upregulated metabolites, and blue dots are downregulated metabolites. (**E**) Network analysis of mouse tissue, comparing CIM and control. Border color and node color: red indicates up-regulation in CIM, and blue indicates down-regulation in CIM. Node size indicates the FDR-adjusted *p*-value.

To discover metabolite changes in skeletal muscle, we used a mouse model induced by injection of immunogens. Mouse quadriceps and hamstrings muscle were stained with hematoxylin and eosin (H&E) to assess muscle histology (Figure 3A). The histological score showed a significantly increased in the quadriceps and hamstring muscles of CIM mice compared to control mice (Figure 3B). CIM showed an increased production of cytokines TNF-α, IL-6, and IL-1 in mouse serum samples (Supplementary Figure S4). We performed univariate and multivariate analyses to explore the effect on the metabolite profile in muscle tissue with inflammation. After missing value imputation, a total of 157 metabolites remained for further analysis in mouse muscle tissue. The PCA analysis in mouse muscle samples showed a difference between CIM and control mice (Figure 3C). A volcano plot was constructed to display the overview of significant metabolites (t-test with FDR-adjusted *p*-value less than 0.05) between CIM and control mice. (Figure 3D). In total, 68 metabolites were changed in the tissue of CIM mice (Supplementary Table S4). A clustered heatmap showed the similarity of the metabolite patterns of individual samples in each group (Supplementary Figure S5). Among significant metabolites, the levels of amino acids, biogenic amines, and acylcarnitines, except taurine, were decreased in CIM mice. Alternatively, the levels of all lipids increased in CIM mice. Interestingly, biogenic amines, except taurine, and all metabolites from other classes showed the same pattern in CIM mice in the network analysis (Figure 3E). In addition, we identified 37 metabolites that were altered in the serum of CIM mice compared to controls (Supplementary Figure S6 and Supplementary Table S5). We noted that metabolites, including carnosine, tryptophan, and creatinine, were altered in the serum of CIM mice (Supplementary Figure S7).

### 3.5. Metabolic Pathway Associated with the Muscle of CIM Mice and Expression of ODC-1 and SMOX

**Figure 4:**
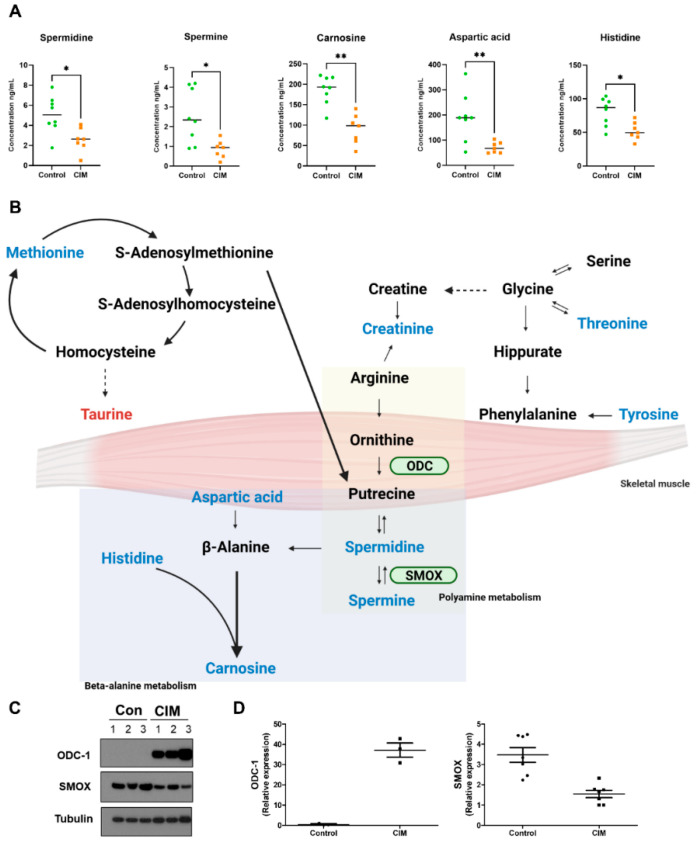
IIM-related metabolite alterations and pathways in muscle tissue. (**A**) Individual scatter plots of significant metabolites related to important pathways in mouse tissue. (**B**) Overview of altered metabolites related to the skeletal muscle tissue of mice. Red-colored metabolite indicates up-regulation in CIM mice, while blue-colored metabolites represent down-regulation as compared with the control mice. (**C**) Protein expression and (**D**) relative quantitative data of ODC-1, SMOX, and tubulin. (* *p* < 0.05, ** *p* < 0.005.)

Following statistical analysis for metabolites that were measured by targeted analysis, enrichment pathway analysis was utilized to recognize the alteration of metabolic pathways that are associated with IIM muscle (Supplementary Figure S8). Pathway analysis showed that 6 metabolites–aspartic acid, histidine, carnosine, spermidine and spermine (Figure 4A)—were predominantly linked to beta-alanine and histidine metabolism (Supplementary Table S6). Furthermore, we found that spermine and spermidine were significantly decreased in the CIM mice, which indicates the down-regulation of the polyamine pathway (Figure 4A,B) as metabolites from the polyamine pathway have been detected in several studies but have not been ascertained in the CIM mouse [14,15]. Thus, we additionally observed the expression of enzymes related to spermine and spermidine. Spermine oxidase (SMOX) converts spermine to spermidine which is a critical regulator of spermine and spermidine concentration, and ornithine decarboxylase 1 (ODC-1) is known as the rate-limiting step of polyamine pathways [16,17]. To further understand the alteration of polyamines concentration in muscle tissue, Western blot analysis was used for ODC-1 and SMOX (Figure 4C,D). The expression of ODC-1 increased, whereas SMOX expression decreased in CIM mice.

## 4. Discussion

Using a targeted metabolomics platform, this study highlights that all groups from patients and healthy control showed different blood metabolic signatures, indicating the potential of the biomarker for IIM and the significant alteration of metabolites to discover the underlying mechanism related to muscle inflammation.

We found notable metabolites, including five amino acids and two biogenic amines besides lipids, that were altered specifically in IIM patients. These findings are in agreement with those of a previous study that showed serum amino acid concentration alteration in IIM patients [13]. Amino acids, especially branched-chain amino acids (BCAA), are essential for skeletal muscle and whole-body metabolism [18]. In previous studies, BCAA is recommended as a supplement for muscle development and treating fatigue [19]. However, increased levels of BCAAs can cause inflammation by activating mTORC1, which subsequently increases the production of proinflammatory cytokines [20]. The observation of the increased BCAA level in the serum in IIM patients needs to be investigated further, along with the levels of other increased amino acids. Remarkably, methionine sulfoxide was specifically increased in IIM comparing healthy controls and AS patients and methionine were decreased in IIM comparing healthy control. Methionine sulfoxide, the oxidized form of methionine, can be reduced back to methionine by methionine reductases that protect proteins against oxidative stress and regulates the aging process [21,22]. An altered level of methionine sulfoxide and methionine is considered a biomarker of oxidative stress, and, therefore, our result might reflect increased ROS production in IIM patients [23,24]. Additionally, our results showed that various metabolites in the lipid class were altered, specifically in IIM patients. Phospholipid metabolism has been highlighted in various diseases and energy metabolisms, as well as in skeletal muscle function [25]. A few studies have reported changes of lipid in serum of myopathy patients and speculated that dysregulation of the lipid metabolism in patients with polymyositis and dermatomyositis results in persistent muscle weakness and fatigue [26,27].

In recent years, machine learning methods have frequently been applied to discover biomarkers of muscle diseases. Previous findings have predicted the cancer risk of anti-TIF1γ+ myositis and have suggested gene markers for muscle disease classification using machine learning algorithms. In this study, we first identified a set of seven metabolites that allowed us to discriminate IIM patients from AS patients and healthy controls by using machine learning algorithms based on the AUC values of LR, RF and SVM were 0.955, 0.908 and 0.918, respectively [28,29]. Our results suggest a prediction biomarker set for IIM patients, which is relevant to the pathophysiologic mechanism of IIM. This biomarker discovery may help for the diagnosis or disease activity monitoring through less invasive and easily applied technologies instead of muscle biopsy.

In the muscle tissue of CIM mice, spermine and spermidine from the polyamine pathway were significantly down-regulated in this study. In addition, ornithine and putrescine, which also are involved in the polyamine pathway, were significantly increased in the serum of IIM patients compared to healthy controls. Dysregulated polyamine metabolism associated with various diseases and inflammation has been described in previous studies [30,31]. Polyamines in muscle disease are related to the degeneration and regeneration of muscular fibers, and the concentrations of spermine and spermidine are regulated by a key enzyme, SMOX [32]. Furthermore, SMOX expression was also reduced, besides the decreased spermine and spermidine concentrations in CIM mice. A recent study demonstrated that a significant reduction of SMOX expression is associated with muscle atrophy induced by several conditions [33]. Aging is one of the conditions that can reduce spermidine levels and SMOX expression, which can bring about a loss of skeletal muscle [34]. The significant reduction of spermine, spermidine, and SMOX expression and significant induction of ODC was highly consistent with that in aged skeletal muscle. These changes in metabolites and enzyme expression might be affected by aging, including decreased cell proliferation and protein synthesis [35]. Thus, the alteration of polyamine metabolites and associated enzymes might reflect an aging phenotype in inflammatory muscle.

Moreover, we discovered changes in metabolites from beta-alanine and histidine metabolisms using enrichment pathway analysis. Compared to the control mice, histidine, carnosine and aspartic acid levels were lower in the muscle tissue of CIM mice. Carnosine, formed by histidine and beta-alanine, is highly presented and synthesized primarily in the skeletal muscle tissue of mammals [36]. In addition, carnosine has protective effects, including the reduction of proinflammatory and profibrotic cytokines, which can suppress ROS production and inflammation [37,38]. Histidine also plays an important role in skeletal muscle that enhances muscle performance and is used for the synthesis of carnosine [39]. Decreased muscle carnosine and histidine may reflect cell damage in states of inflammation.

In our results, interestingly, metabolic profiling in tissue samples from CIM mice showed metabolites, except taurine, from each class changed in the same direction. The significant reduction in several amino acids and amines, such as aspartic acid, histidine, methionine, threonine, tyrosine, carnosine, creatinine, spermidine, and spermine that was observed in our study, is highly consistent with previous studies with aging. Essential amino acids stimulate protein synthesis in skeletal muscle, and the delivery of amino acids is closely associated with muscle weakness and atrophy [40]. Previous studies further identified that reduced amino acid delivery and protein synthesis is stimulated by mTORC1 in aged skeletal muscle [41]. Our findings in this study showed particular relevance to the aging phenotype.

The limitations of the present study include the lack of a validation cohort for modeling, the small sample size of patient groups and healthy controls, the lack of various disease controls, the potential gender difference of polyamine metabolites in the mouse model and only partial metabolites representation of the blood and tissue metabolomes. The lack of a validation set in this study could lead to the risk of model overfitting. As IIM is a rare disease, additional validation cohort recruitment was challenging. To overcome this limitation, we developed the five-fold cross-validation method with three machine learning algorithms. The limitation of the targeted analysis was that only a set of metabolites could be evaluated. As various metabolites in our study showed significant differences in IIM and the mouse model, further research using global profiling is needed.

## 5. Conclusions

In summary, we performed targeted metabolomics profiling in the serum of IIM patients and tissue of CIM mice and showed a specific metabolic signature within both serum and inflamed tissue. To the best of our knowledge, this is the first study suggesting a metabolic biomarker for IIM patients by using a machine learning algorithm-based prediction model. Beyond suggesting a predictive marker of IIM, our study additionally uncovered alteration of metabolites related to muscle inflammation and provided new insight into IIM pathology.

## Figures and Tables

**Table 1:** Demographics and clinical parameters of study participants.

	IIM (*n* = 50)	AS (*n* = 30)	Control (*n* = 10)	*p*-Value
Age (year) *	50.7 ± 12.2	52.5 ± 11.4	45.0 ± 15.0	0.249
Male	25 (50.0)	15 (50.0)	5 (50.0)	1.000
BMI (kg/m^2^)	23.5 ± 3.4	22.8 ± 2.8	25.0 ± 2.2	0.376
Age at disease onset (year)	48.4 ± 12.4			
Disease duration (year)	2.24 ± 2.74			
Clinical features at disease onset (*n*)				
Proximal muscle weakness	34 (68.0)			
Skin manifestations †	30 (60.0)			
Interstitial lung disease	27 (55.1)			
Elevated serum level of enzymes ‡	46 (92.0)			
Anti-Jo1 antibody present	6 (12.2)			
Laboratory data *				
WBC (×10^3^/μL)	8445 ± 5095			
Creatinine kinase (U/L)	532 ± 934			
Aldolase (U/L)	19.1 ± 23.2			
Myoglobin (ng/mL)	694 ± 885			
LDH (IU/L)	329 ± 179			
C-reactive protein (mg/dL)	1.44 ± 3.59			
ESR (mm/h)	30.5 ± 28.1			
Treatment history ^§^				
Corticosteroids (ever)	41 (82.0)			
IVIG (ever)	4 (8.0)			

Note: Values are expressed as the mean ± SD or numbers (%) unless stated otherwise. * At sample collection date; † Gottron’s sign or heliotrope rash or V-neck sign; ‡ Creatinine kinase or lactate dehydrogenase or aspartate aminotransferase or alanine aminotransferase; ^§^ Treatment history before the sample collection date; Abbreviation: ANA, Antinuclear antibodies; ESR, Erythrocyte sedimentation rate; IVIG, Intravenous immunoglobulin; LDH, Lactate dehydrogenase; WBC, White blood cell.

## Data Availability

This data is available at the NIH Common Fund’s National Metabolomics Data Repository (NMDR) website, the Metabolomics Workbench, https://www.metabolomicsworkbench.org (accessed on 14 July 2022), where it has been assigned Project ID PR001400. The data can be accessed directly via its Project DOI: 10.21228/M8RM5T. This work is supported by NIH grant U2C-DK119886.

## Supplementary Materials

The following supporting information can be downloaded at: https://www.mdpi.com/article/10.3390/metabo12101004/s1, Table S1. A detailed list of all metabolites.; Table S2. List of significant metabolites in human serum. Statistical analysis was performed using one-way analysis of variance (ANOVA).; Table S3. List of IIM-specific metabolites.; Table S4. List of significant metabolites in mouse tissue. Statistical analysis was performed using a *t*-test.; Table S5. List of significant metabolites in mouse serum. Statistical analysis was performed using a *t*-test; Table S6. Significantly altered pathway using enrichment analysis in mouse tissue.; Figure S1. Study workflow.; Figure S2. Cluster heatmap for significant metabolites in IIM. (**A**) including amino acid, biogenic amine, acylcarnitine class and (**B**) SMs, lysoPCs, PC, and hexoses.; Figure S3. ROC curves and AUC values of a set of 7 metabolites panel in the prediction model for discriminating IIM patients.; Figure S4. Increased production of cytokines TNF-α, IL-6, and IL-1 in CIM compared to control.; Figure S5. Heatmap of significant metabolites with all mice skeletal muscle samples.; Figure S6. Metabolic profiles in mouse serum samples. (**A**) PCA plot of control and CIM skeletal muscle serum. (**B**) Volcano plot from t-test results of control and CIM. (**C**) Heatmap of significant metabolites with all mice serum samples.; Figure S7. MetaMapp metabolomic networks of mouse serum altered in CIM compared to control. Border color and node color: red indicates up-regulation in IIM or CIM-vehicle, and blue indicates down-regulation in IIM or CIM-vehicle. Node size indicates FDR-adjusted *p*-value.; Figure S8. Pathway enrichment dot plot.
