# Supplementary material for: Identification of Metabolic Signature Associated with Idiopathic Inflammatory Myopathy Reveals Polyamine Pathway Alteration in Muscle Tissue
Source: Metabolites. 2022 Oct 21;12(10):1004. doi: 10.3390/metabo12101004 (PMC9611268; doi:10.3390/metabo12101004)

SUPPLEMENTARY MATERIALS

Supplementary Table and Figure Legends

Table S1. Detailed list of all metabolites.

Table S2. List of significant metabolites in human serum. Statistical analysis was performed using one-way analysis of variance (ANOVA).

Table S3. List of IIM specific metabolites.

Table S4. List of significant metabolites in mouse tissue. Statistical analysis was performed using t-test.

Table S5. List of significant metabolites in mouse serum. Statistical analysis was performed using t-test.

Table S6. Significantly altered pathway using enrichment analysis in mouse tissue.

Figure S1. Study workflow

Figure S2. Cluster heatmap for significant metabolites in IIM. A, including amino acid, biogenic amine, acylcarnitine class and B, SMs, lysoPCs, PC, and hexoses.

Figure S3. ROC curves and AUC values of a set of 7 metabolites panel in prediction model for discriminating IIM patients.

Figure S4. Increased production of cytokines TNF-α, IL-6, and IL-1 in CIM compared to control.

Figure S5. Heatmap of significant metabolites with all mice skeletal muscle samples.

Figure S6. Metabolic profiles in mouse serum samples. A, PCA plot of control and CIM skeletal muscle serum. B, Volcano plot from t-test results of control and CIM. C, Heatmap of significant metabolites with all mice serum samples.

Figure S7. MetaMapp metabolomic networks of mouse serum altered in CIM compared to control. Border color and node color: red indicates up-regulation in IIM or CIM-vehicle, blue indicates down-regulation in IIM or CIM-vehicle. Node size indicates FDR-adjusted p-value.

Figure S8. Pathway enrichment dot plot.

Table S1. List of significant metabolites in human serum. Statistical analysis was performed using one-way analysis of variance (ANOVA).

Metabolites	FDR-adjusted p-value
Met-SO	4.49E-06
PC aa C38:4	1.55E-05
Glu	3.55E-05
PC ae C38:0	8.07E-05
PC aa C38:6	8.07E-05
PC aa C38:1	8.07E-05
PC ae C36:1	8.07E-05
PC aa C42:4	8.58E-05
PC ae C38:6	8.58E-05
PC aa C40:6	8.58E-05
PC aa C24:0	0.0001
lysoPC a C24:0	0.0002
PC ae C38:2	0.0002
PC ae C38:5	0.0002
PC aa C36:4	0.0003
Gln	0.0003
Kynurenine	0.0003
C2	0.0003
PC ae C36:5	0.0003
PC aa C38:5	0.0003
PC aa C42:5	0.0005
PC aa C36:0	0.0005
PC ae C42:4	0.0006
PC ae C40:5	0.0006
PC aa C40:3	0.0006
lysoPC a C26:0	0.0006
PC aa C42:2	0.0009

PC ae C30:2	0.0009
Serotonin	0.0010
Asp	0.0011
PC aa C40:1	0.0011
PC aa C40:2	0.0021
Met	0.0022
Phe	0.0024
lysoPC a C20:4	0.0026
PC ae C42:5	0.0026
PC ae C40:6	0.0026
Ile	0.0026
Ala	0.0026
Leu	0.0026
PC ae C44:3	0.0026
Ser	0.0027
ADMA	0.0027
PC ae C44:4	0.0027
SM C26:0	0.0037
Pro	0.0039
SM (OH) C22:2	0.0040
PC ae C36:4	0.0040
C14:1	0.0040
lysoPC a C28:0	0.0040
lysoPC a C17:0	0.0041
Orn	0.0041
PC ae C30:0	0.0042
PC aa C42:1	0.0042
PC ae C40:2	0.0042
Lys	0.0042
PC ae C38:3	0.0042
PC ae C42:3	0.0043
PC aa C30:2	0.0043
lysoPC a C28:1	0.0043
PC aa C40:4	0.0052
Val	0.0057
SM (OH) C22:1	0.0061
PC ae C40:4	0.0063
PC aa C38:3	0.0077
PC ae C30:1	0.0094
Creatinine	0.0100

Taurine	0.0112
Tyr	0.0112
Gly	0.0135
PC ae C38:0	0.0151
lysoPC a C16:0	0.0151
alpha-AAA	0.0151
PC ae C40:3	0.0169
SM (OH) C24:1	0.0170
Putrescine	0.0173
PC ae C36:2	0.0173
PC ae C42:1	0.0179
PC aa C40:5	0.0183
PC ae C42:2	0.0232
PC aa C32:0	0.0308
PC aa C42:6	0.0330
t4-OH-Pro	0.0333
lysoPC a C18:0	0.0361
lysoPC a C16:1	0.0422
SM (OH) C14:1	0.0430
PC aa C36:3	0.0439
PC ae C36:0	0.0439

Table S2. Detailed list of all metabolites.

Metabolite abbreviation	Full name	HMDB ID
Ala	L-Alanine	HMDB0000161
Arg	L-Arginine	HMDB0000517
Asn	L-Asparagine	HMDB0000168
Asp	L-Aspartic acid	HMDB0000191
Cit	Citrulline	HMDB0000904
Gln	L-Glutamine	HMDB0000641
Glu	L-Glutamic acid	HMDB0000148
Gly	Glycine	HMDB0000123
His	L-Histidine	HMDB0000177
Ile	L-Isoleucine	HMDB0000172
Leu	L-Leucine	HMDB0000687

Lys	L-Lysine	HMDB0000182
Met	L-Methionine	HMDB0000696
Orn	Ornithine	HMDB0000214
Phe	L-Phenylalanine	HMDB0000159
Pro	L-Proline	HMDB0000162
Ser	L-Serine	HMDB0000187
Thr	L-Threonine	HMDB0000167
Trp	L-Tryptophan	HMDB0000929
Tyr	L-Tyrosine	HMDB0000158
Val	L-Valine	HMDB0000883
Ac-Orn	N-Acetylornithine	HMDB0003357
ADMA	Asymmetric dimethylarginine	HMDB0001539
alpha-AAA	Aminoadipic acid	HMDB0000510
c4-OH-Pro	cis-4-Hydroxyproline	HMDB0240251
Carnosine	Carnosine	HMDB0000033
Creatinine	Creatinine	HMDB0000562
DOPA	L-Dopa	HMDB0000181
Dopamine	Dopamine	HMDB0000073
Histamine	Histamine	HMDB0000870
Kynurenine	L-Kynurenine	HMDB0000684
Met-SO	Methionine sulfoxide	HMDB0002005
Nitro-Tyr	3-Nitrotyrosine	HMDB0001904
PEA	Phenylethylamine	HMDB0012275
Putrescine	Putrescine	HMDB0001414
SDMA	Symmetric dimethylarginine	HMDB0003334
Serotonin	Serotonin	HMDB0000259
Spermidine	Spermidine	HMDB0001257
Spermine	Spermine	HMDB0001256
t4-OH-Pro	4-Hydroxyproline	HMDB0000725
Taurine	Taurine	HMDB0000251

Table S3. List of IIM specific metabolites.

Metabolite	IIM	AS	Healthy control	ANOVA(FDR-adjusted p-value)	Post-hoc analysis
Ala	723.84±46.99	566.23±43.33	480.5±36.72	0.0026	**, †
Asp	157.89±19.58	82.03±14.32	44.66±3.84	0.0011	***, †
Glu	650.62±46.49	397.02±51.95	242.4±33.14	0.0000	****, †††
Orn	224.1±16.49	162.05±11.65	130.57±9.14	0.0041	**, †
Val	406.22±26.74	330.53±24.58	291.9±12.19	0.0057	*, †
Met-SO	39.36±4.47	22.42±7.25	2.3±0.44	0.0000	****, †††
Serotonin	0.31±0.02	0.48±0.05	0.56±0.08	0.0010	**, ††
lysoPC a C20:4	4.54±0.23	5.53±0.28	6.75±0.72	0.0026	**, †
lysoPC a C24:0	0.53±0.03	0.33±0.04	0.16±0.05	0.0002	***, ††
lysoPC a C26:0	0.76±0.08	0.5±0.09	0.16±0.03	0.0006	***, †
PC aa C24:0	0.31±0.02	0.17±0.02	0.1±0.02	0.0001	***, †††
PC aa C36:0	6.84±0.46	4.68±0.4	3.72±0.39	0.0005	**, ††
PC aa C36:4	76.98±3.22	102.41±5.25	111.26±9.39	0.0003	**, †††
PC aa C38:1	4.68±0.37	2.54±0.42	2.4±0.37	0.0001	*, ††††
PC aa C38:4	41.43±1.71	64.67±3.91	68.59±6.37	0.0000	***, ††††
PC aa C38:5	24.48±1.35	36.09±2.74	39.76±3.87	0.0003	**, †††
PC aa C38:6	48.14±2.57	67±5.12	88.33±4.36	0.0001	****, ††
PC aa C40:3	2.46±0.16	1.68±0.18	1.11±0.16	0.0006	***, †
PC aa C40:4	4.94±0.26	3.75±0.25	3.44±0.47	0.0052	*, ††
PC aa C40:6	18.82±1.09	29.84±2.68	32.73±1.76	0.0001	***, †††
PC aa C42:1	0.48±0.03	0.37±0.03	0.32±0.03	0.0042	*, †
PC aa C42:2	0.9±0.05	0.59±0.05	0.55±0.07	0.0009	*, †††
PC aa C42:4	1.3±0.08	0.79±0.08	0.59±0.1	0.0001	***, †††
PC aa C42:5	0.73±0.03	0.52±0.04	0.5±0.06	0.0005	*, †††
PC ae C30:2	0.25±0.01	0.16±0.02	0.14±0.02	0.0009	**, ††

PC ae C36:1	22.43±1.44	13.97±1.5	9.73±0.85	0.0001	***, †††
PC ae C36:4	8.55±0.49	11.1±0.8	12.3±1.04	0.0040	**, †
PC ae C36:5	5.53±0.31	7.55±0.5	8.86±0.77	0.0003	***, ††
PC ae C38:0	9.63±0.77	5.43±0.82	3.01±0.44	0.0001	****, †††
PC ae C38:2	9.41±0.64	6.12±0.82	3.68±0.47	0.0002	***, ††
PC ae C38:3	13.71±0.77	9.76±1.1	8.55±1.37	0.0042	*, ††
PC ae C38:5	7.22±0.37	9.94±0.65	11.52±1.12	0.0002	**, †††
PC ae C38:6	3.9±0.25	5.71±0.47	6.66±0.4	0.0001	***, †††
PC ae C42:3	1.85±0.09	1.4±0.11	1.21±0.14	0.0043	*, †
PC ae C42:4	1.76±0.08	1.23±0.09	1.29±0.15	0.0006	*, †††
PC ae C44:4	0.53±0.02	0.44±0.02	0.36±0.03	0.0027	**, †
SM (OH) C24:1	0.95±0.04	1.08±0.05	1.15±0.08	0.0170	*, †

Concentration of each group represented as mean ± SEM. Comparing Healthy control vs IIM, * p <0.05; ** p < 0.005; *** p < 0.0005, *** p < 0.0001. Comparing AS vs IIM, † p <0.05; †† p < 0.005; ††† p < 0.0005, †††† p < 0.0001. Listed metabolites were not significantly different between healthy control and AS.

Table S4. List of significant metabolites in mouse tissue. Statistical analysis was performed using t-test.

Metabolites	Fold change	FDR-adjusted p-value
PC ae C36:2	3.1	0.0122
PC ae C34:2	3.1	0.0122
PC ae C36:3	3.0	0.0122
PC aa C34:3	2.8	0.0122
PC aa C36:2	3.5	0.0139
PC aa C34:2	2.4	0.0139
Creatinine	0.5	0.0139
PC aa C36:1	3.5	0.0171
PC aa C40:4	3.2	0.0171
PC aa C32:0	2.9	0.0171
PC ae C34:3	2.8	0.0171
PC aa C34:1	2.6	0.0171
PC aa C32:2	2.6	0.0171
PC ae C34:1	2.6	0.0171
PC ae C38:5	2.5	0.0171
PC ae C36:4	2.3	0.0171
SM C16:0	2.1	0.0171
Carnosine	0.5	0.0171
C0	0.5	0.0171
SM (OH) C24:1	1.7	0.0171
PC ae C36:5	3.1	0.0195
PC aa C40:5	3.1	0.0195
PC aa C36:3	2.7	0.0195
PC aa C36:0	2.6	0.0195
PC ae C32:1	2.4	0.0195
PC ae C36:1	2.4	0.0195
PC aa C32:1	2.3	0.0195
C18:1	0.5	0.0195
PC ae C30:1	1.8	0.0195
Taurine	1.1	0.0195
C18:1-OH	0.6	0.0228
C16:1	0.4	0.0238
PC aa C38:4	2.5	0.0238
PC ae C38:2	2.4	0.0238
PC ae C34:0	2.3	0.0238

PC aa C38:1	2.2	0.0238
PC ae C32:2	2.2	0.0238
PC aa C42:4	1.9	0.0238
SM (OH) C16:1	1.8	0.0238
PC aa C38:0	2.5	0.0255
PC aa C40:2	2.5	0.0283
C18:2	0.3	0.0293
SM C24:1	2.8	0.0293
SM (OH) C22:1	2.7	0.0293
PC aa C38:3	2.4	0.0293
PC aa C40:6	2.4	0.0293
PC ae C38:6	2.2	0.0293
PC ae C42:1	2.0	0.0293
Met	0.7	0.0293
Tyr	0.7	0.0293
PC ae C30:0	3.0	0.0313
PC aa C30:2	1.6	0.0359
Asp	0.4	0.0364
PC aa C42:5	1.9	0.0364
PC ae C36:0	1.8	0.0364
Spermine	0.3	0.0372
Spermidine	0.5	0.0372
PC ae C38:4	1.9	0.0372
His	0.6	0.0372
SM (OH) C22:2	2.3	0.0460
SM C24:0	2.8	0.0474
C14:1	0.5	0.0474
SM (OH) C14:1	1.9	0.0474
C16:1-OH	0.6	0.0474
PC ae C38:0	1.6	0.0474
C2	0.6	0.0474
Thr	0.7	0.0474
PC ae C40:4	1.3	0.0474

Table S5. List of significant metabolites in mouse serum. Statistical analysis was performed using t-test.

Metabolites	Fold change	FDR-adusted p-value
lysoPC a C20:4	0.3	3.E-05
lysoPC a C20:3	0.5	0.0002
t4-OH-Pro	1.7	0.0004
Trp	0.6	0.0005
lysoPC a C17:0	0.6	0.0007
PC ae C34:1	1.5	0.0007
lysoPC a C18:2	0.5	0.0007
Carnosine	0.3	0.0007
lysoPC a C18:1	0.6	0.0010
lysoPC a C16:0	0.7	0.0012
Arg	1.7	0.0012
PC aa C40:2	1.8	0.0016
lysoPC a C16:1	0.7	0.0034
H1	0.6	0.0036
PC aa C42:4	1.6	0.0038
PC ae C40:1	0.6	0.0038
lysoPC a C18:0	0.7	0.0038
ADMA	1.6	0.0043
PC aa C36:0	0.7	0.0049
SM C26:0	0.6	0.0067
PC ae C38:0	0.7	0.0078
PC ae C36:5	0.7	0.0093
SM C18:0	0.7	0.0110
PC aa C32:1	1.5	0.0175
PC aa C30:0	1.2	0.0184
SM C16:0	1.3	0.0216
SM C24:1	1.3	0.0216
PC ae C32:1	1.3	0.0244
PC aa C36:6	0.8	0.0277
lysoPC a C24:0	0.6	0.0279
PC aa C36:4	0.6	0.0279
SM C18:1	0.7	0.0344
PC ae C42:3	0.7	0.0458
C0	0.8	0.0458
Creatinine	0.8	0.0458
SM (OH) C22:2	0.8	0.0458
PC aa C38:4	0.6	0.0488

Table S6. Significantly altered pathway using enrichment analysis in mouse tissue.

Pathway	Total	Hit	Metabolites	P-value
Beta-Alanine metabolism	21	5	L-Aspartic acid, Carnosine, Spermine, L-Histidine, Spermidine	0.0018
Histidine metabolism	16	4	L-Histidine, L-Aspartic acid, Carnosine	0.0315

Figure S1. Study workflow

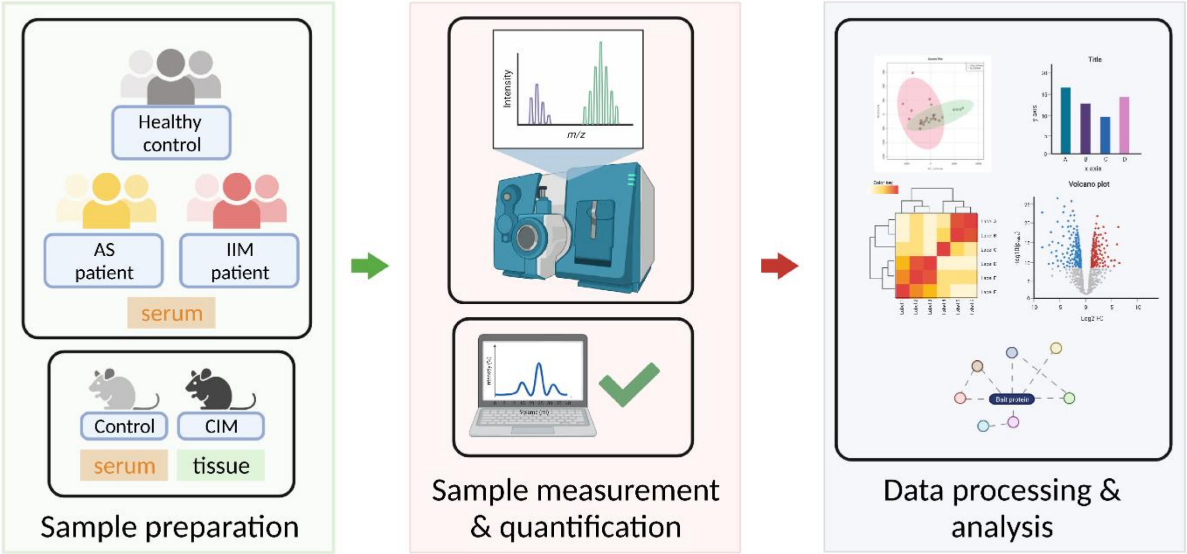

Figure S2. Cluster heatmap for significant metabolites in IIM. A, including amino acid, biogenic amine, acylcarnitine class and B, SMs, lysoPCs, PC, and hexoses.

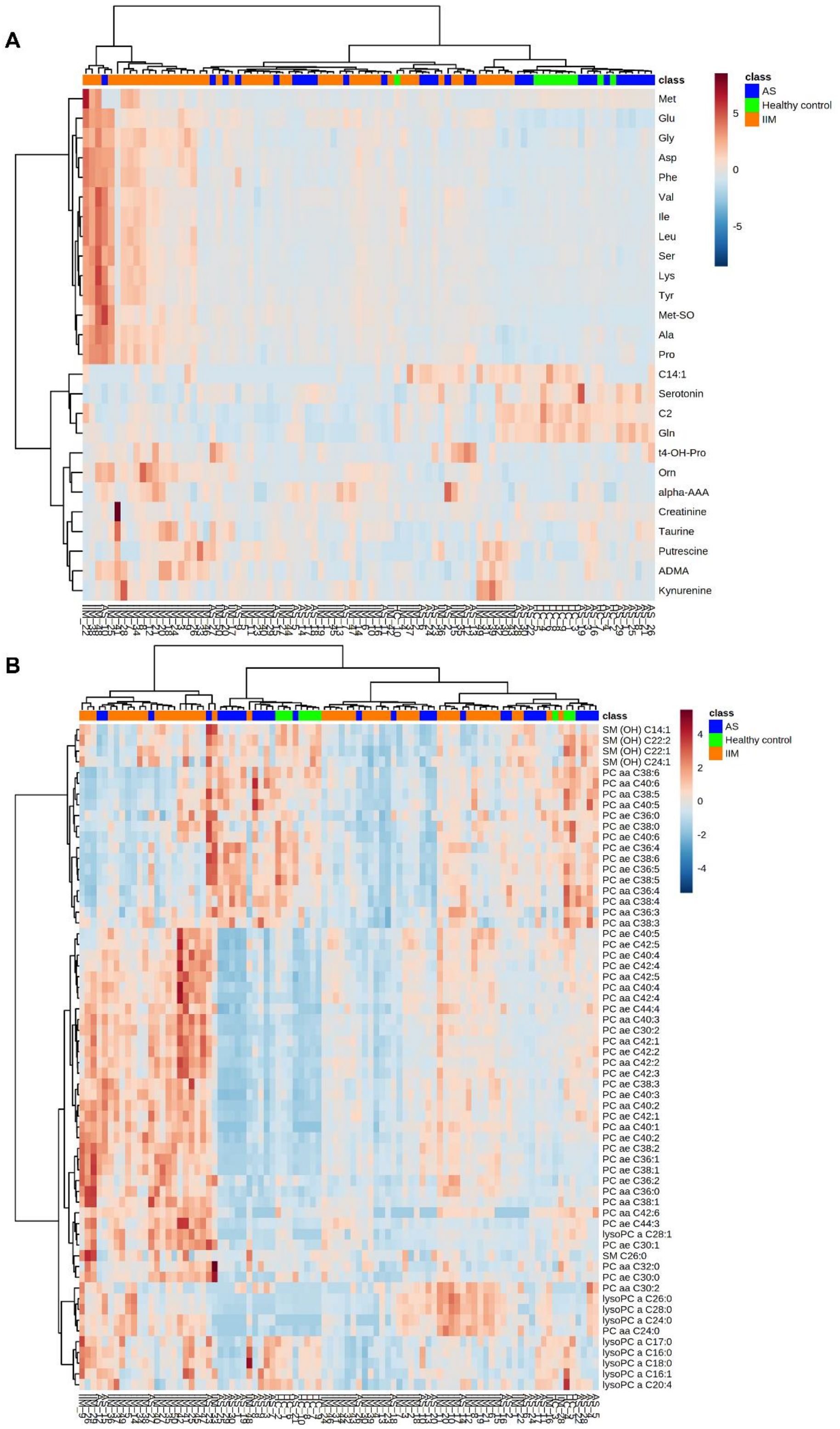

Figure S3. ROC curves and AUC values of a set of 7 metabolites panel in prediction model for discriminating IIM patients.

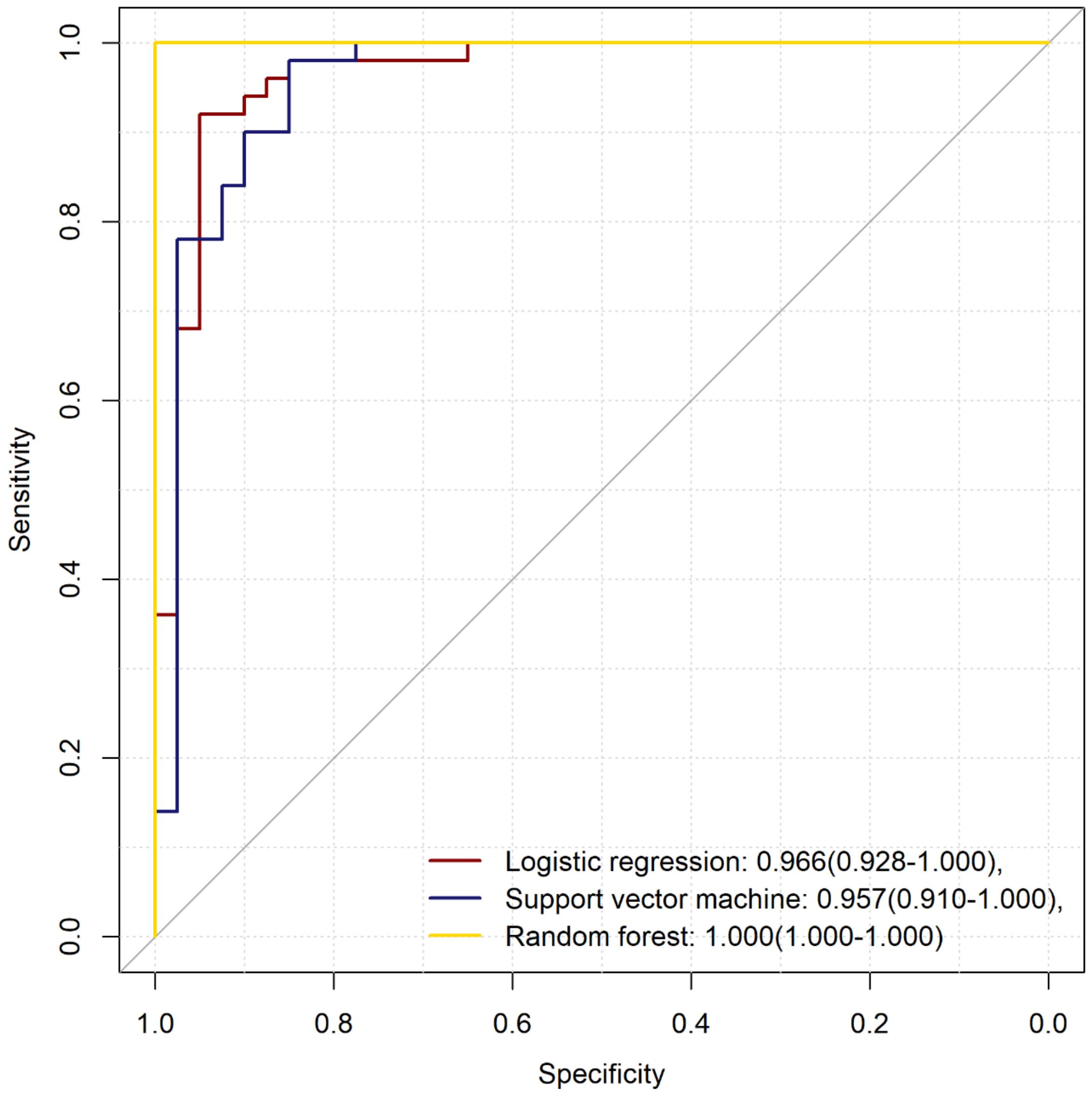

Figure S4. Increased production of cytokines TNF-α, IL-6, and IL-1 in serum of mouse model.

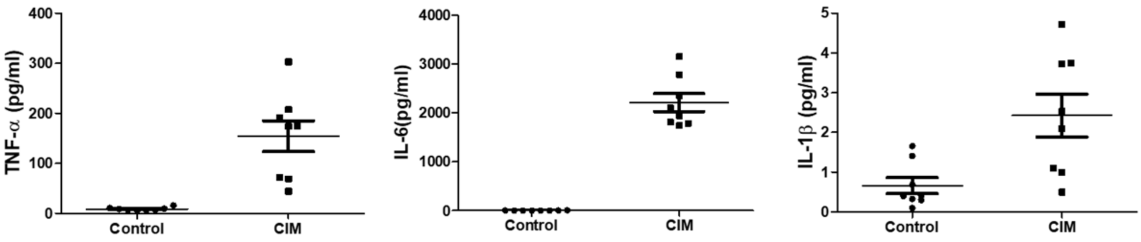

Figure S5. Heatmap of significant metabolites with all mice skeletal muscle samples.

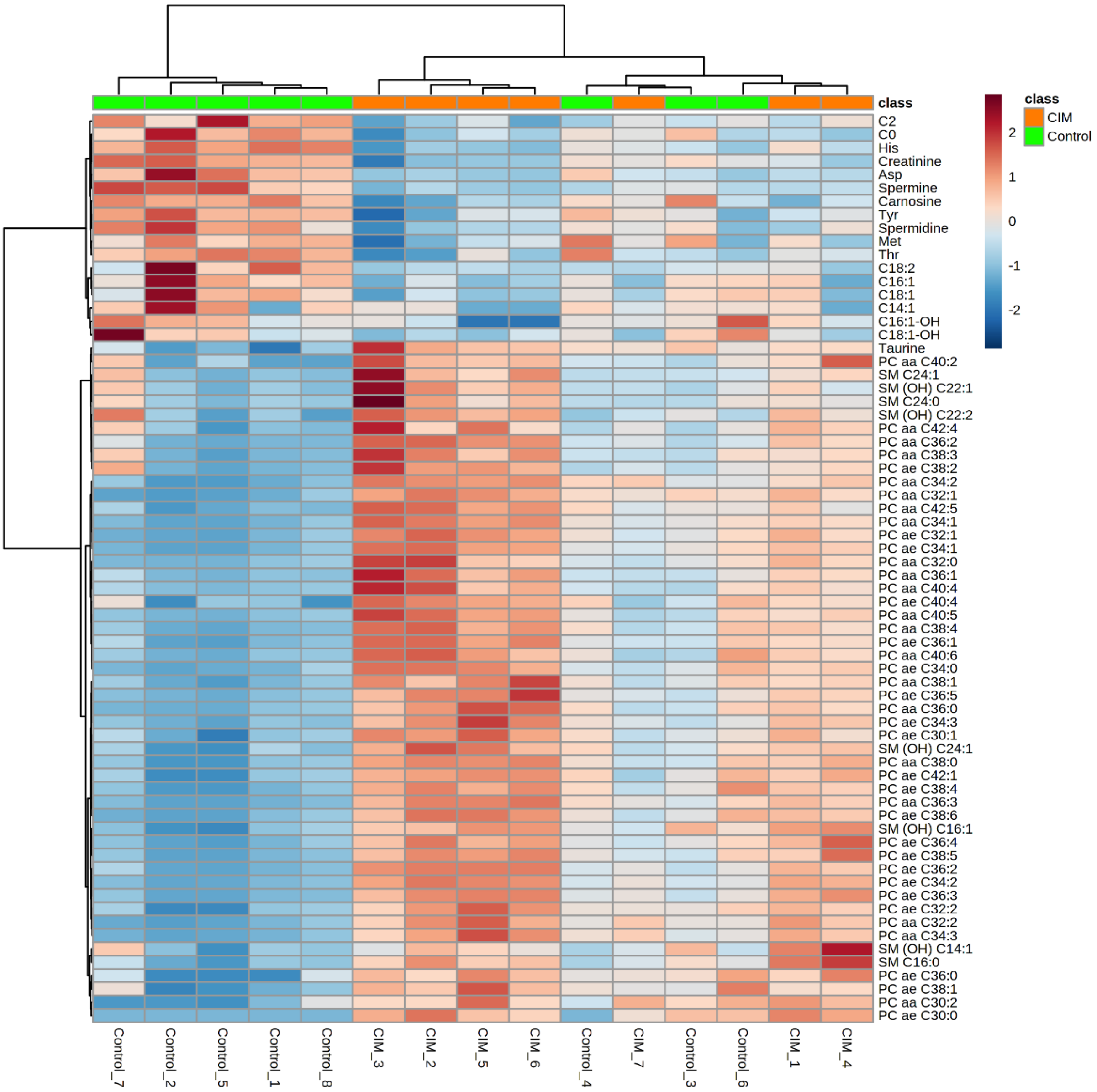

Figure S6. Metabolic profiles in mouse serum samples. A, PCA plot of control and CIM skeletal muscle serum. B, Volcano plot from t-test results of control and CIM. C, Heatmap of significant metabolites with all mice serum samples.

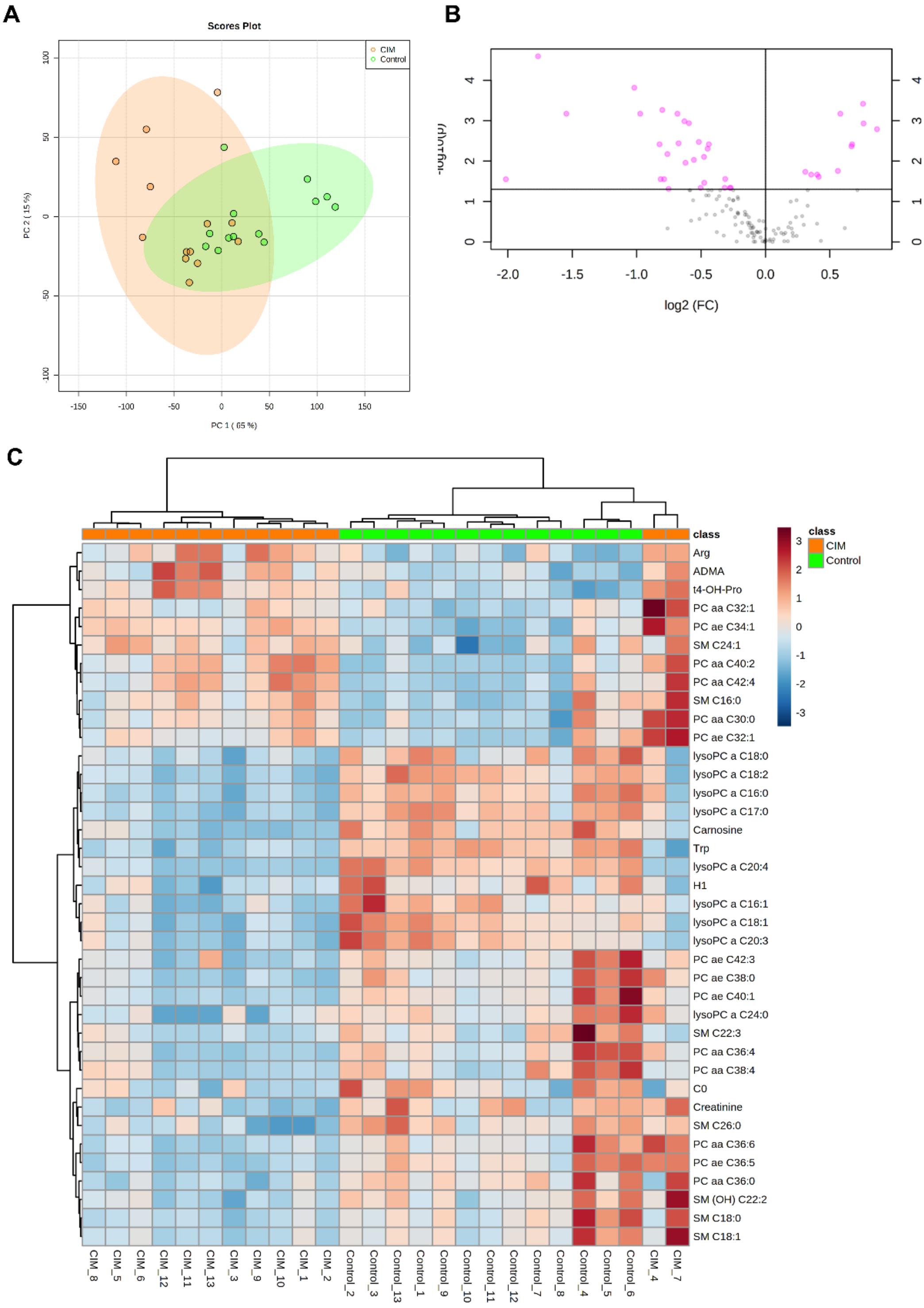

Figure S7. MetaMapp metabolomic networks of mouse serum altered in CIM compared to control. Border color and node color: red indicates up-regulation in CIM, blue indicates down-regulation in IIM. Node size indicates FDR-adjusted p-value.

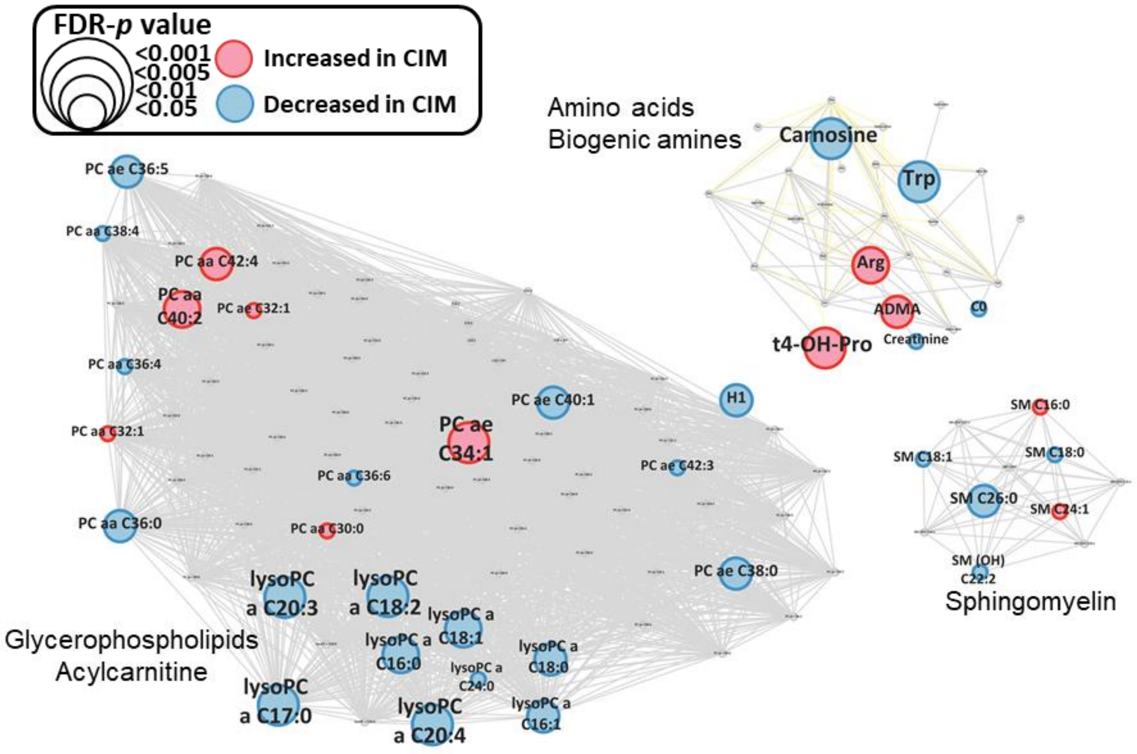

Figure S8. Pathway enrichment dot plot.

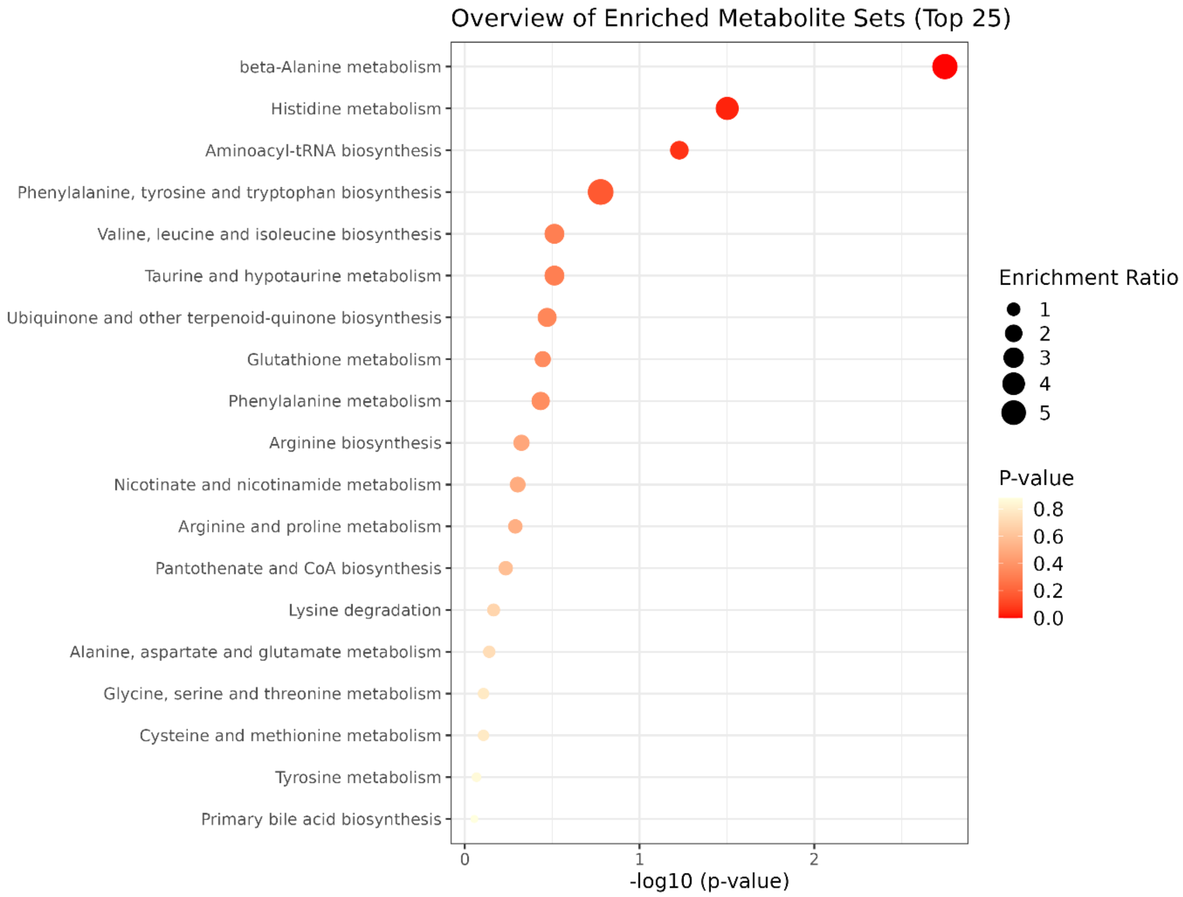

Supplement: Supplementary file 1 [file metabolites-12-01004-s001.zip › metabolites-1968144-supplementary.pdf]
